# Solvent‐Free Supramolecular Polymerization for Feather‐Like Nanostructured Chiral Fluorescent Polyurethanes with Multimodal Chiroptical Stimuli Responsiveness

**DOI:** 10.1002/advs.202417572

**Published:** 2025-02-28

**Authors:** Huimin Duan, Shuli Li, Xinlei Wu, Jianping Deng, Jiawei Li, Dongming Qi, Biao Zhao

**Affiliations:** ^1^ School of Textile Science and Engineering, School of Materials Science and Engineering & School of Chemistry and Chemical Engineering Zhejiang Sci‐Tech University Zhejiang 310018 China; ^2^ State Key Laboratory of Chemical Resource Engineering, College of Materials Science and Engineering Beijing University of Chemical Technology Beijing 100029 China; ^3^ Zhejiang Provincial Innovation Center of Advanced Textile Technology Zhejiang 312000 China; ^4^ Shaoxing Keqiao Research Institute of Zhejiang Sci‐Tech University Zhejiang 312000 China

**Keywords:** chiral fluorescent polyurethanes, circularly polarized luminescence, feather‐like nanostructure, solvent‐free supramolecular polymerization, stimuli responsiveness

## Abstract

Chiral supramolecular polymers with stimuli‐responsive circularly polarized luminescence (CPL) are highly desirable for smart flexible optoelectronic devices, but remain rarely reported. Here, a simple solvent‐free supramolecular polymerization for preparing chiral polyurethanes is presented by in situ induced self‐assembly strategy, using cellulose nanocrystals (CNCs)‐based isocyanate prepolymers and macromolecular polyols as precursors, achieving precise control over polymer chain assembly with spot‐like arrangement. More importantly, by further incorporating a π‐conjugated luminescent dihydroxynaphthalene molecule, CPL‐active flexible polyurethane films with feather‐like nanostructures are constructed, which promote the ordered arrangement of CNCs‐based isocyanate segments due to the increased spatial resistance. The π─H bond network between CNCs and urethane‐linked benzene rings drives the self‐assembly, enabling higher‐level chiral amplification and enhanced fluorescence. Interestingly, the prepared chiral fluorescent polyurethanes display multimodal chiroptical stimuli responsiveness under various stimuli, such as temperature, solvent polarity, pH, and polarized light, due to the sensitivity of the π─H bond network. This work offers new insights into designing solvent‐free chiral supramolecular polymers with significant chiroptical potentials.

## Graphical abstract

Precise construction of the feather‐like nanostructured chiroptical polyurethanes using cellulose nanocrystals (CNCs)‐based isocyanate prepolymers with fluorescent naphthalene structures and macromolecular polyols as precursors is manifested by solvent‐free supramolecular polymerization. Furthermore, the sensitivity of the π─H bond network to various stimuli, including temperature, solvent polarity, pH, and polarized light, is applied to realize multimodal chiroptical stimuli responsiveness.

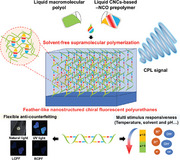

## 1 Introduction

Circularly polarized luminescence (CPL),^[^ ^1^ ^]^ a unique optical phenomenon where the electric field vector of emitted light helically rotates during propagation, has gained significant attention for its potential applications in 3D display technologies,^[^ ^2^ ^]^ optical data storage,^[^ ^3^ ^]^ and smart sensing.^[^ ^4^ ^]^ Chiral supramolecular assemblies (CSAs),^[^ ^5^ ^]^ with their exceptional chiral amplification capabilities and dynamic responsiveness, exhibit enhanced molecular recognition accuracy, higher storage density, and stronger interference resistance in intelligent sensing and information storage applications, compared to existing technologies, making them strong candidates for high‐performance CPL materials. Over the past years, CSAs have been extensively studied under different solvent conditions, and their mechanisms have been well elucidated.^[^ ^6^ ^]^ However, certain solvent systems can directly affect the chiral retention and self‐assembly behavior of CSAs.^[^ ^7^ ^]^ If CSAs could be constructed under solvent‐free conditions, the resulting multi‐level superstructures could be directly utilized without compromising their structural integrity.^[^ ^8^ ^]^ More importantly, solvent‐free system can avoid the potential pollution problems, which is more advantageous from the viewpoint of practical applications. Nevertheless, under solvent‐free conditions, the reduced diffusion rates, increased molecular motion resistance, high rotational barriers and changes in intermolecular interactions make it difficult to precisely control the size and morphology of molecular assemblies, thereby increasing the difficulty of achieving well‐ordered structures through self‐assembly.^[^ ^9^ ^]^ To date, controllable construction of ordered CSAs in solvent‐free environments remains a significant challenge.

Solvent‐free reactive polyurethanes (RPU), prepared via in‐situ polymerization of isocyanate‐capped prepolymers and macromolecular polyols, are ideal candidates for constructing functional supramolecular assemblies due to their highly designable and controllable structures.^[^ ^10^ ^]^ On the other hand, cellulose nanocrystals (CNCs), due to their intriguing hierarchical chirality and abundant hydroxyl and charged groups on the surface, which can form covalent and non‐covalent bonds with achiral luminophores,^[^ ^11^ ^]^ are considered as powerful chiral platform compound for constructing CPL‐active materials. Judiciously incorporating CNCs into the RPU main chain via urethane linkages is expected to fabricate ordered structures of chiral supramolecular polyurethanes (CSPs), building up a new class of advanced chiroptical materials. In addition, the inherent characteristics of RPU including superior mechanical strength, high thermal stability and good processibility of RPU can ensure the resulting CSPs meeting different application scenarios. However, to our knowledge, there have been no example of integrating RPU and CNCs to construct CPL materials.

**Scheme 1:**
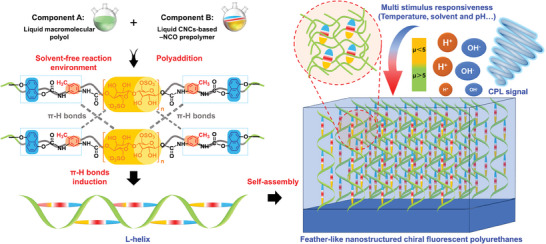
Schematic depicting the preparation of chiral fluorescent polyurethanes (Poly‐CNCs_X_‐DN) with multimodal chiroptical stimuli responsiveness via solvent‐free supramolecular polymerization.

In this study, we developed a simple solvent‐free supramolecular polymerization for preparing a series of CPL‐active CSPs (named as Poly‐CNCs_X_‐DN) by in situ‐induced self‐assembly strategy, using CNCs‐based isocyanate prepolymers, π‐conjugated 1,5‐dihydroxynaphthalene (1,5‐DN) and liquid macromolecular polyols as precursors (**Scheme** **1**). Detailed structural characterizations demonstrate the π─H bonds network between CNCs and urethane‐linked benzene rings drives the formation of feather‐like nanostructures, enabling higher‐level chiral amplification and enhanced fluorescence in the synthesized CSPs. More interestingly, due to the sensitivity of the π─H bonds network, the prepared CSPs display multimodal chiroptical stimuli responsiveness under various stimuli, such as temperature, solvent polarity, pH, and polarized light. Additionally, the potential application of the obtained CSPs in multiple anti‐counterfeiting was demonstrated.

## 2 Results and Discussion

### 2.1 Solvent‐Free Supramolecular Polymerization for Chiral Fluorescent RPU

Cellulose nanocrystals (CNCs) with high crystallinity, uniform size, and abundant sulfate ester groups were prepared by sulfuric acid hydrolysis.^[^ ^12^ ^]^ CNCs‐based chiral NCO‐terminated prepolymers were successfully synthesized by polyaddition of 2,4‐toluene diisocyanate (TDI) with polytetrahydrofuran ether diol (PTMEG‐1000) and varying CNCs ratios (1%, 2%, 4%, and 6%) at 80 °C under a nitrogen atmosphere. The prepolymer method was employed to synthesize the solvent‐free reactive polyurethanes (RPU), where the formation of a chiral fluorescent ‐NCO terminated prepolymer was followed by chain extension to gradually construct hard segments. This approach enhanced the formation of a more favorable and ordered microphase‐separated structure.^[^ ^13^ ^]^ The side reactions were minimized by controlling the reaction rate through reduced reactivity. Additionally, the ─NCO to polyol ratio was flexibly adjusted, improving the compatibility between CNCs and the RPU backbone. Detailed procedures for the synthesis and characterization of the CNCs and prepolymers were provided in the Supporting Information (Figures S1–S5 and Table S1, Supporting Information). A series of chiral supramolecular RPU (Poly‐CNCs_X_‐DN; _X_ represents the content of CNCs) were synthesized through a solvent‐free method using TDI, PTMEG‐1000, CNCs and 1,5‐dihydroxy naphthalene (1,5‐DN) as monomers. The CNCs serve as the chiral source, while 1,5‐DN provides the fluorescent properties, as schematically illustrated in Scheme 1. Besides, pure polyurethane in the absence of both CNCs and 1,5‐DN (named Pure‐poly) and achiral luminescent polyurethane containing 1,5‐DN (named Poly‐DN) were also prepared as control experiments.

**Figure 1:**
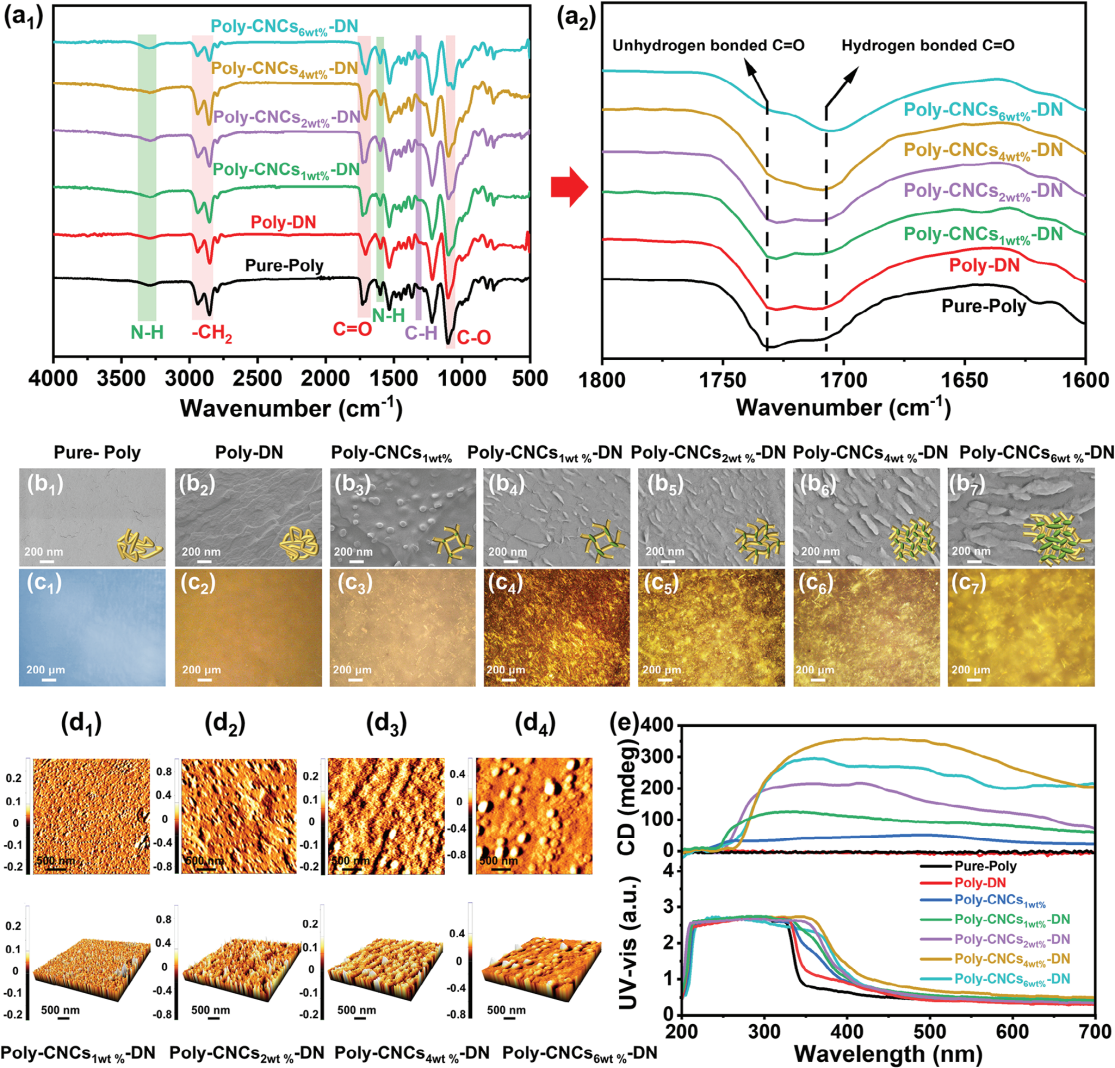
Microstructure and optical activity. ATR‐FTIR spectra (a_1_, 500–4000 cm^−1^, a_2_, 1600–1800 cm^−1^), b) SEM images and corresponding schematic diagram cross‐linked network structure, POM images c), AFM images d), and CD and UV‐vis absorption spectra e) of Pure‐Poly, Poly‐DN, Poly‐CNCs_X,_ and Poly‐CNCs_X_‐DN films.

To confirm the successful synthesis of Poly‐CNCs_X_‐DN, attenuated total reflection Fourier transform infrared (ATR‐FTIR) spectra were obtained (**Figure** **1**a). For Pure‐Poly, the mian RPU characteristic bands included N─H stretching at 3300 cm⁻¹, C═O stretching at 1730 cm⁻¹, N─H bending at 1530 cm⁻¹, and C─O ether at 1110 cm⁻¹ (Figure 1a_1_).^[^ ^14^ ^]^ The absorption bands at 2854 and 2930 cm⁻¹ corresponded to the symmetric and asymmetric C─H stretching vibrations of ─CH_2_ groups,^[^ ^15^ ^]^ and the C─H stretching vibration peak at 1311 cm⁻¹ was attributed to the phenyl group of TDI. The absence of ─NCO peak (2260–2280 cm⁻¹) indicated complete consumption, while hydrogen‐bonded and free C═O peaks (1700 and 1730 cm⁻¹, respectively) further approved the synthesis of Pure‐Poly.^[^ ^16^ ^]^ In Poly‐DN films, the absence of ‐OH peaks (3600 and 1270 cm⁻¹) and changes in C═O peaks suggested the formation of π‐hydrogen bonding (π─H bond).^[^ ^17^ ^]^ Shifts in C─O ether (1110 cm⁻¹) and N─H bending (1530 cm⁻¹) indicated bonding between 1,5‐DN and polyurethane.^[^ ^18^ ^]^ In Poly‐CNCsx‐DN films, with the increase of CNCs content, the further change of C═O and C─O peaks demonstrated that new hydrogen bonds between CNCs and polyurethane. Besides, the red‐shift of the ‐C─O stretching vibration peak at 1110 cm⁻¹, coupled with the significant enhancement of the N─H bending vibration peak at 1530 cm⁻¹ in Poly‐CNCs_X_‐DN films, indicates the covalent attachment of CNCs into the polyurethane main chain.^[^ ^19^ ^]^ The Poly‐CNCs_X_‐DN films exhibited excellent weather resistance due to enhanced crosslinking from CNCs,^[^ ^20^ ^]^ making them insoluble in most solvents (Figures S6 and S7, Supporting Information).

The molecular morphology and orientation of the prepared Poly‐CNCs_X_‐DN films were characterized by scanning electron microscopy (SEM) and polarizing optical microscopy (POM). In the solvent‐free system, both Pure‐Poly (Figure 1b_1_,c_1_) and Poly‐DN (Figure 1b_2_,c_2_) films exhibited smooth surfaces without ordered morphology or orientation. When 1 wt.% CNCs were introduced into the RPU main chain, the Poly‐CNCs_1wt.%_ film formed ordered structures with a spot‐like arrangement and birefringence, owing to CNCs aggregation and self‐assembly into ordered microregions (Figure 1b_3_,c_3_). Further, Poly‐CNCs_1wt.%_‐DN film (Figures 1b_4_,c_4_) with a more regular, feather‐like morphology and enhanced birefringence was obtained by incorporating both CNCs and 1,5‐DN, compared to Poly‐CNCs_1wt.%_ film. This suggested that the naphthalene ring's extensive conjugated electron cloud in 1,5‐DN facilitated a higher degree of ordered orientation in CNCs aggregates.^[^ ^21^ ^]^ By maintaining the 1,5‐DN content constant and increasing the CNCs content to 2 wt.%, the Poly‐CNCs_2wt.%_‐DN film exhibited a denser feather‐like morphology and more significant birefringence (Figures 1b_5_,c_5_), confirming CNCs as the primary inducer of regular nanostructures. Higher CNCs levels (4 and 6 wt.%) resulted in more complex, densely stacked feather‐like morphologies and crystal birefringence in the Poly‐CNCs_4wt.%_‐DN and Poly‐CNCs_6wt.%_‐DN films (Figures 1b_6‐7_,c_6‐7_), indicating that excessive CNCs disrupt the formation and orderliness of nanocrystalline phases in polyurethane.^[^ ^22^ ^]^

Due to the thermodynamic exclusion principle,^[^ ^23^ ^]^ the formation of ordered nanocrystalline phases in the segments also promoted the ordered phase separation between hard and soft segments, as shown in atomic force microscopy (AFM) images of Poly‐CNCs_1wt.%_‐DN (Figure 1d_1_), Poly‐CNCs_2wt.%_‐DN (Figure 1d_2_), and Poly‐CNCs_4wt.%_‐DN (Figure 1d_3_) films. However, as the CNCs content increased from 4 to 6 wt.%, the surface roughness and characteristic feature size of the Poly‐CNCs_6wt.%_‐DN significantly rose (Figure 1d_4_), with prominent aggregates and a rough texture becoming more apparent. The SEM images further revealed substantial aggregation of CNCs, forming large clusters and an uneven distribution. These results suggested that an excessive increase in CNCs concentration affected the orderliness of microphase separation between soft and hard segments in RPU.

The circular dichroism (CD) and ultraviolet (UV)‐visible (vis) absorption spectra of Poly‐CNCs_X_‐DN films were analyzed to understand the impact of CNCs and 1,5‐DN incorporating into the RPU main chain on the optical activity, as shown in Figure 1e. The CD spectra of Pure‐Poly and Poly‐DN exhibited nearly none optical activity across the entire wavelength range. However, the introduction of 1,5‐DN into RPU gave rise to a visible red‐shift in UV‐vis absorption compared to Pure‐Poly films, attributed to π─π interactions that stabilize the excited state and lower the transition energy.^[^ ^24^ ^]^ When 1 wt.% CNCs were incorporated into RPU, red‐shifted UV‐vis absorption (230–700 nm) and positive CD signals emerged, indicating that CNCs self‐assemble into ≈50 nm dot‐like morphologies and introduce secondary chirality (Figure 1b_3_). This self‐assembly formed ordered crystalline regions in the hard segments, promoting microphase separation (Figure 1d) and tertiary chiral assembly, enhancing optical activity. This high‐level chiral structure imparted optical activity and induced CD signals to the RPU. Interestingly, by adding 1,5‐DN to the Poly‐CNCs_1wt.%_ film, the CD signals of the Poly‐CNCs_1wt.%_‐DN film were further enhanced, and UV‐vis absorption was significantly red‐shifted from 335 to 350 nm, indicating that 1,5‐DN promoted chiral amplification of CNCs in RPU.^[^ ^25^ ^]^ Besides, SEM (Figure 1b_4_) and AFM (Figure 1d_1_) analyses revealed an interesting feather‐like morphology (≈200 nm) and extended microphase separation, suggesting unique interactions between 1,5‐DN and CNCs, which induced aggregation of hard segments into layered crystals, achieving high‐level chiral amplification under solvent‐free conditions. Furthermore, the enhancement of the CD signal in Poly‐CNCs_X_‐DN film and the red‐shift of UV‐vis absorption above 350 nm became evident with the increase in CNCs content. This suggested that CNCs acted as chiral sources for high‐level self‐assembly, making them the primary factor responsible for inducing chirality in RPU. Thus, the Poly‐CNCs_4wt.%_‐DN film showed the strongest optical activity, demonstrating optimal synergy between CNCs‐based segments and 1,5‐DN. Conversely, increasing the CNCs content to 6 wt.% in Poly‐CNCs_6wt.%_‐DN film resulted in decreased CD intensity, due to excessive CNCs aggregation disrupting optimal molecular arrangement and reducing chiral transfer efficiency.

Based on the above discussions, the formation of dot‐like nanostructures was primarily due to the self‐assembly of CNCs within the RPU chains, creating hard segment aggregates that propagate chirality throughout the RPU backbone. Further, the feather‐like nanostructures resulted from the steric hindrance introduced by the 1,5‐DN naphthalene ring and its extensive conjugated electron cloud, which promoted the ordered alignment of CNCs aggregates. Coupled with the urethane linkage between CNCs and naphthalene rings, larger and more long‐range ordered hard‐segment aggregates were formed, enhancing optical activity. Additionally, the assembled morphology of CNCs exhibits layered alignment,^[^ ^11^ ^]^ which facilitates the formation of feather‐like structures.

### 2.2 Chiral Amplification Mechanism

**Figure 2:**
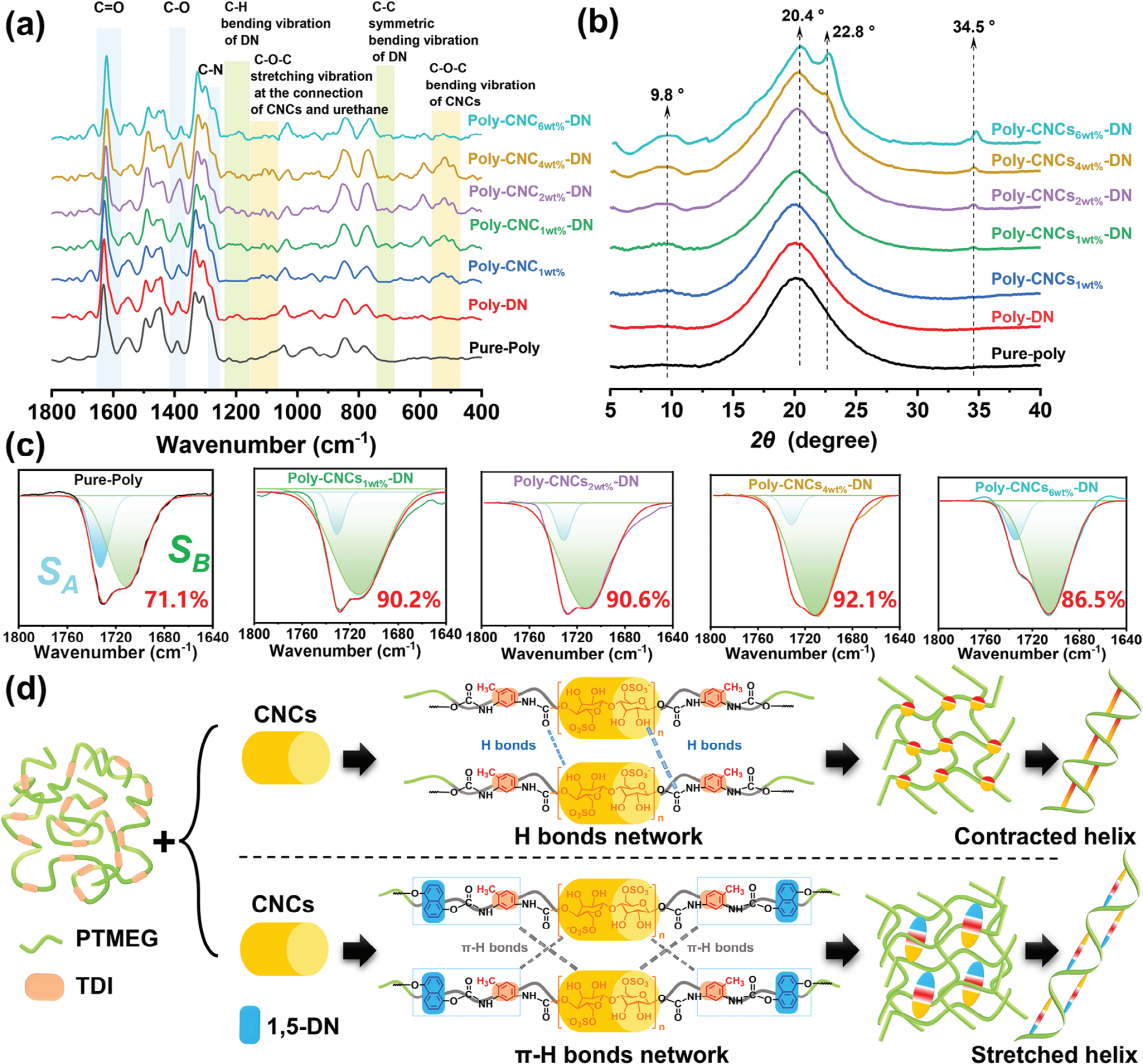
Crystallization driving force. Raman spectra a) and XRD pattern b) of Pure‐Poly, Poly‐DN, Poly‐CNCs_X_ and Poly‐CNCs_X_‐DN films. ATR‐FTIR spectra c, 1640–1780 cm^−1^) of Pure‐Poly and Poly‐CNCs_X_‐DN films. Schematic diagram d) of the chiral amplification mechanism.

To investigate the chiral amplification mechanism in the synthesized chiral supramolecular RPU films, we examined the crystallization driving force of CNCs‐based hard segments owing to the close connection between the chiral amplification and driving force, using Raman, ATR‐FTIR, and XRD characterizations.^[^ ^26^ ^]^ Initially, the Raman spectrum (**Figure** **2**a) of the Pure‐Poly film displayed characteristic peaks at 1620 cm⁻¹ (C═O stretching), 1390 cm⁻¹ (C─O stretching), and 1270 cm⁻¹ (C─N stretching) of urethane groups.^[^ ^26a,b^ ^]^ In the Poly‐DN film, these peaks were shifted and intensified, with additional peaks at 1190 and 700 cm⁻¹, attributed to C─H and C─C bending of 1,5‐DN. This indicated strong π─H bonds interactions between π‐electrons of 1,5‐DN and the hydrogen donors (N─H groups) within the RPU chains. Subsequently, the Poly‐CNCs_1wt.%_ film showed red‐shifts at 1620, 1390, and 1270 cm⁻¹, suggesting enhanced hydrogen bonding. New peaks at 1110 and 520 cm⁻¹ corresponded to C─O─C stretching vibrations at the connection CNCs and urethane, and C─O─C bending vibrations of CNCs, respectively, indicating improved molecular alignment for stable helices. Furthermore, the Poly‐CNCs_1wt.%_‐DN film demonstrated pronounced synergistic effects from CNCs and 1,5‐DN. Peaks at 1190 and 700 cm⁻¹ indicated strong π‐π interactions. The enhancement of π‐H interactions were indicated by noticeable shifts and increased intensities at 1620, 1390, and 1270 cm⁻¹. Increased backbone flexibility and stability were suggested by the peak at 520 cm⁻¹. As CNCs content increased to 2 and 4 wt.%, the Raman spectra showed enhanced π─H bond interactions, with more obvious peaks observed at 1190, 1110, 700, and 520 cm⁻¹. The obvious shifts and increased intensities at 1620, 1390, and 1270 cm⁻¹ indicated robust π‐H networks and stable helical conformations. However, the Poly‐CNCs_6wt.%_‐DN film displayed decreased peaks at 1110 and 520 cm⁻¹, indicating excessive CNCs compromised chain flexibility and helical stability.

The XRD patterns (Figure 2b) of Pure‐poly, Poly‐DN, Poly‐CNCs_1wt.%_, Poly‐CNCs_1wt.%_‐DN, Poly‐CNCs_2wt.%_‐DN, Poly‐CNCs_4wt.%_‐DN, and Poly‐CNCs_6wt.%_‐DN films, were analyzed to elucidate their crystalline structures and helical conformations, focusing on characteristic peaks observed at 2θ ≈ 9.8, 20.4, 22.8, and 34.5°.^[^ ^26c,d^ ^]^ In Pure‐poly and Poly‐DN films, a broad peak at 2θ ≈ 20.4° indicated an amorphous structure lacking long‐range order. The Poly‐CNCs_1wt.%_ film showed a new peak at 2θ ≈ 9.8°, associated with long‐period stacking, suggesting α‐helix‐like structures with a large pitch introduced by CNCs. In the Poly‐CNCs_1wt.%_‐DN film, the peak at 2θ ≈ 9.8° was more pronounced, reflecting the synergistic effect of CNCs and 1,5‐DN's in enhancing molecular packing and order. The observation of new peaks at 22.8 and 34.5°, corresponding to the (200) and (003) planes of a hexagonal columnar structure, indicated tighter hard segment packing and increased crystallinity, possibly initiating π‐helix conformations. As CNCs content increased, peaks at 9.8, 22.8, and 34.5° strengthen, indicating greater crystallinity and order. The peaks at 9.8, 22.8, and 34.5° represented the presence of long‐period stacking, tight molecular packing, and highly ordered crystalline structure. This demonstrated that CNCs and 1,5‐DN enhanced crystalline structure and molecular order, leading to highly ordered, potentially hexagonal columnar configurations. However, in Poly‐CNCs_6wt.%_‐DN films, peaks at 22.8 and 34.5° shifted to higher angles, suggesting reduced interplanar spacing from tighter chain packing. Excessive crystallinity limited chain rotation and reduced optical activity. This analysis aligned with SEM and CD data (Figure 1b,e).

Raman and XRD analyses revealed that π─H bonds play a crucial role in the formation of long‐range ordered structures, characterized by hexagonal columnar crystals and helical RPU chains, leading to a mixed α‐helix and π‐helix configuration. The chiral amplification mechanism was further confirmed using the hydrogen bond index (*HBI*) derived from ATR‐FTIR spectroscopy with Gaussian‐Lorentz peak fitting (Figure 2c).^[^ ^26e,f^ ^]^ ATR‐FTIR spectra focused on the carbonyl (C═O) stretching region (1700─1720 cm⁻¹), with the A peak indicating non‐hydrogen bonded carbonyls and the B peak representing hydrogen‐bonded carbonyls, including π─H bonds. Pure‐Poly had an *HBI* of 71.1%, reflecting N─H…O interactions. In contrast, Poly‐CNCs_1wt.%_‐DN film exhibited a significant increase to 90.2% *HBI*, attributed to π‐electron conjugation from 1,5‐DN and new O─H…O bonding sites from CNCs, enhancing π─H bonds networks. Increasing CNCs content raised the *HBI* to 90.6% for Poly‐CNCs_2wt.%_‐DN film and 92.1% for Poly‐CNCs_4wt.%_‐DN film, indicating stronger π─H bonds interactions. Poly‐CNCs_4wt.%_‐DN film achieved the highest *HBI*, reflecting an optimal CNCs and 1,5‐DN concentration for an efficient π─H bonds network. At 6 wt.% CNCs, the *HBI* declined to 86.5%, suggesting excessive CNCs disrupted the π─H bonds network.

Based on the above data analysis, a schematic diagram of the chiral amplification mechanism was illustrated in Figure 2d. CNCs serve as a secondary chiral source with crystallization properties, transferring chirality to the RPU main chain via chemical bonds. The ordered hard segment aggregates were induced during this process through intermolecular O─H…O hydrogen bonding, resulting in a dot‐like morphology and α‐helical conformation (Poly‐CNCs_1wt.%_) with a contracted helix. Furthermore, 1,5‐DN with a π‐conjugated structure was chemically bonded to the RPU backbone, introducing steric hindrance and forming intramolecular π─H bonds between the urethane's N─H groups and the π‐electrons of 1,5‐DN. These intramolecular π─H bonds facilitated the formation of intermolecular π─H bonds with CNCs, establishing a continuous chiral transfer bridge. Consequently, a feather‐like layered structure was observed of Poly‐CNCs_X_‐DN films, characterized by hexagonal columnar crystals and a π‐helical conformation with a stretched helix.

### 2.3 Multimodal Chiroptical Stimuli Responsiveness

**Figure 3:**
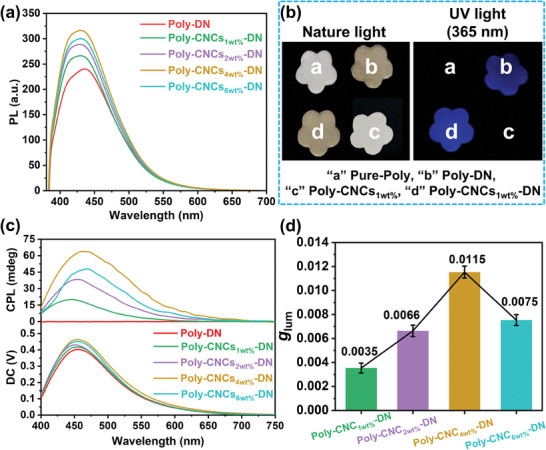
Activation of CPL activity. Photoluminescence (PL) spectra a), Fluorescent images b, *λ*_ex_ = 365 nm), CPL spectra c, *λ*_ex_ = 365 nm), and *g*_lum_ values d) of Pure‐Poly, Poly‐DN, Poly‐CNCs_X_ and Poly‐CNCs_X_‐DN films.

The introduction of fluorescent 1,5‐DN units into the main chain of RPU endowed the resulting polymers with typical photoluminescence (PL) performance. As shown in **Figure** **3**a,b, both Poly‐CNCs_X_‐DN and Poly‐DN films exhibited intense blue fluorescence centered at ≈450 nm. More importantly, compared with Poly‐DN film, the fluorescence intensity, quantum yield (*Φ*_F_), and fluorescence lifetime (*τ*) of Poly‐CNCs_X_‐DN film significantly increased (Table S2, Supporting Information), as the enhanced crystallinity and rigidity of the chiral RPU restricted intramolecular vibrations and rotations, reducing non‐radiative decay and allowing for greater energy emission.^[^ ^27^ ^]^ Besides, the enhanced crystallinity also provided greater spatial hindrance, limiting the aggregation of 1,5‐DN and reducing aggregation‐induced fluorescence quenching effect.

In view of the chirality and fluorescence behaviors of the prepared chiral supramolecular RPU, their circularly polarized luminescence (CPL) properties were further recorded. As presented in Figure 3c, strong CPL emissions at ≈460 nm were clearly detected in Poly‐CNCs_X_‐DN films. As the CNCs content increased, the intensity of CPL signals strengthened and reached the maximum in Poly‐CNCs_4wt.%_‐DN, indicating enhanced chiral transmission due to more effective π─H bonds networks and ordered structures. This enhancement resulted from CNCs forming intermolecular π─H bonds networks with 1,5‐DN in the RPU main chain, effectively transmitting and amplifying the chiral signal. Besides, the CPL signal of the Poly‐CNCs_4wt.%_‐DN film at various geometric angles (0, 90, 180, and 270°) kept consistent, confirming the reliability (Figure S8, Supporting Information).^[^ ^28^ ^]^ Besides, due to the absence of chiral factor, no CPL signal was detected in Poly‐DN film, further confirming the necessity of CNCs for CPL emission in the present system. Moreover, the luminescence dissymmetry factor (|*g*_lum_|) values followed the same trend (Figure 3d; Table S2, Supporting Information), with |*g*_lum_| value for Poly‐CNC_1wt.%_‐DN, Poly‐CNC_2wt.%_‐DN, Poly‐CNC_4wt.%_‐DN, and Poly‐CNC_6wt.%_‐DN films being 0.0035, 0.0066, 0.0115, and 0.0075, respectively. Additionally, as the CNCs content increased, a red‐shift in the CPL signal occurred due to the formation of supramolecular chiral structures with larger effective ordering dimensions. As shown in Table S2 (Supporting Information), the red‐shift in PL emission arose from the enhanced π─H bonds, which stabilized the excited state and reduced the transition energy of the Poly‐CNC_X_‐DN film.

The generation of CPL was attributed to two main factors: first, CNCs and 1,5‐DN were linked by urethane bonds, forming π─H bonds network between RPU molecules. This network served as an ideal bridge for the transmission and amplification of chiral signals. Secondly, CNCs enhanced the crystallinity and rigidity of the RPU, reducing intramolecular vibrations, non‐radiative decay, and aggregation‐induced fluorescence quenching of 1,5‐DN. These factors collectively contributed to the increase of fluorescence intensity, resulting in a strong CPL effect. Besides, the CPL brightness (*B*_CPL_, defined as *B*_CPL_ = *ε_λ_* × *Φ*_F_ ×[|*g*_lum_|]/2) of Poly‐CNCs_X_‐DN films were further calculated to quantitatively evaluate the overall efficiency of circularly polarized emitters, as listed in Table S2 (Supporting Information). Poly‐CNCs_4wt.%_‐DN film showed largest *B*_CPL_ = 18.22 value compared to other Poly‐CNCs_X_‐DN films. The above results demonstrated that the obtained films exhibited intense CPL activity. Additionally, Figure S9 (Supporting Information) showed the overlap between the CD band of the chiral Poly‐CNCs_1wt.%_ film and the emission of the fluorescent Poly‐DN film, indicating that Poly‐CNCs_1wt.%_ film acted as a handed‐selective fluorescence filter, converting achiral fluorescence molecule into circularly polarized light.^[^ ^29^ ^]^

**Figure 4:**
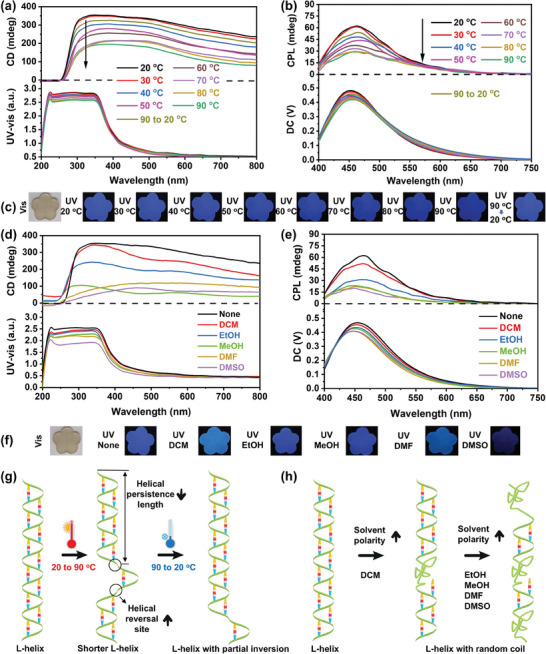
Multimodal stimuli responsiveness on temperature and solvent polarity. CD and UV‐vis absorption spectra a, d), CPL spectra b, e, *λ*_ex_ = 365 nm), Fluorescent images c, f) and Schematics g, h) illustrating changes in helical conformation and interaction forces of Poly‐CNCs_4wt.%_‐DN film with respect to varying temperature and solvent, respectively.

The stimulus‐responsive behavior of chiral supramolecular RPU was investigated to understand their potential applications in advanced optical and electronic devices, sensors, and smart materials. Taking Poly‐CNCs_4wt.%_‐DN film as a representative, its response to different external stimulus were studied. First, we explored the effect of temperature on Poly‐CNCs_X_‐DN films. As the temperature increased from 20 to 90 °C, the CD intensity of Poly‐CNCs_4wt.%_‐DN film at 350 nm decreased by 51.6% (**Figure** **4**a), indicating a loss of chiral order likely due to thermal disruption of polymer chain alignment.^[^ ^30^ ^]^ Interestingly, upon cooling back to 20 °C, the CD strength returned to 83.4% of its original intensity, suggesting partial reversibility in chiral supramolecular assembly. These results demonstrated the temperature sensitivity of the CD effects in Poly‐CNCs_4wt.%_‐DN film and its partial reversible helical memory. Correspondingly, the CPL spectra (Figure 4b) and *g*_lum_ value (Table S3, Supporting Information) showed that CPL intensity, particularly ≈450 nm, decreased with rising temperature, reflecting the loss of excited‐state chirality. Upon cooling, the CPL signal recovered 80% of its original intensity, reinforcing the reversible nature of these thermal effects. This was consistent with the CD results. The PL signal exhibited a slight decrease in intensity with increasing temperature ≈450 nm (Figure 4b,c), indicating thermal sensitivity of the electronic properties. The electronic properties and basic structure of 1,5‐DN, being stable aromatic rings, were typically not significantly affected within the 20 to 90 °C range. The decrease in fluorescence intensity with increasing temperature pointed to diminished emission efficiency due to increased polymer chain mobility, weakening π─H bonds interactions between the naphthalene ring and urethane groups, and affecting electronic excited‐state energy transfer. This temperature‐dependent behavior in CPL performance was attributed to the disruption of stabilizing interactions by thermal energy, including hydrogen bonding and π─H bonds, leading to reduced structural order and less efficient electronic transitions (Figure 4g). These findings highlighted the thermal sensitivity and dynamic adaptability of chiral and electronic properties in chiral supramolecular RPU, emphasizing their potential for applications in smart materials and responsive optical devices.

Solvent polarity response on CD, CPL, and PL properties of Poly‐CNCs_4wt.%_‐DN film were further explored (Figure 4d–f). After wetting with DCM, the initial CD signal of the Poly‐CNCs_4wt.%_‐DN film was slightly reduced (Figure 4d), while the chiral structure was preserved. Further CD signal reductions occurred with EtOH, MeOH, DMF, and DMSO, accompanied by UV‐vis absorption peaks blue‐shifting, indicating the appearance of a random‐coil segment on the main chain.^[^ ^31^ ^]^ The CPL spectra demonstrated significant responsiveness of the Poly‐CNCs_4wt.%_‐DN film to solvent polarity (Figure 4e; Table S4, Supporting Information). The CPL intensity of the Poly‐CNCs_4wt.%_‐DN film was reduced by 31.3% with DCM treatment, while reductions of 55.6% and 65.2% were observed with EtOH and MeOH, respectively. Furthermore, DMF and DMSO caused additional reductions in CPL intensity of 66.9% and 72.2%, accompanied by pronounced blue‐shifts. Optical images (Figure 4f) followed a similar trend. These reductions in intensity and the blue‐shifts were attributed to the formation of robust solvation shells around the hydrogen bond donors and acceptors in the Poly‐CNCs_4wt.%_‐DN film when exposed to highly polar solvents, weakening π‐H interactions and disrupting the chiral structure, thus decreasing helical persistence length (Figure 4h).

**Figure 5:**
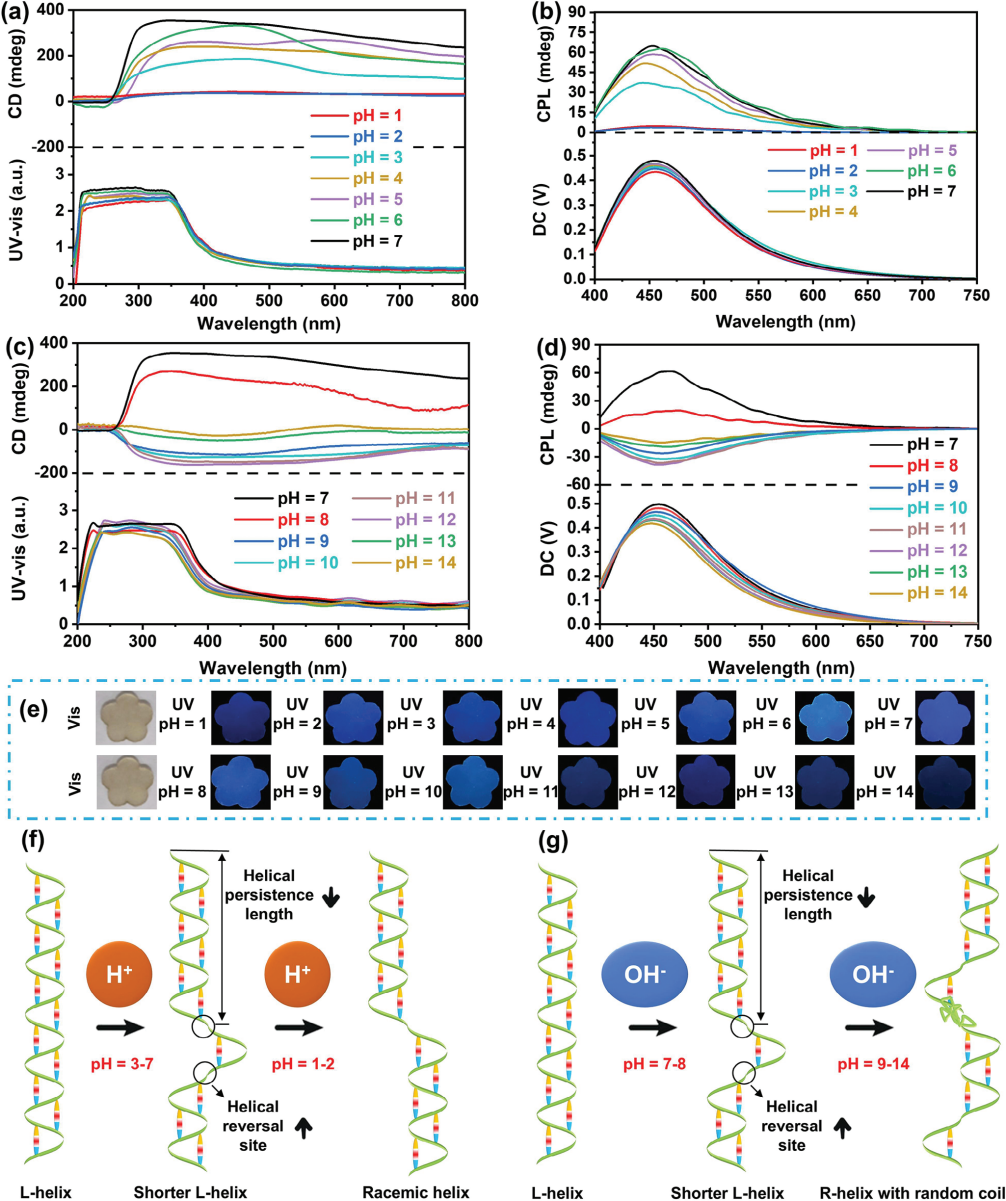
Multimodal stimuli responsiveness on pH values. CD and UV‐vis absorption spectra a, c), CPL spectra b, d, *λ*_ex_ = 365 nm, Fluorescent images e) and Schematics f, g) illustrating changes in helical conformation and interaction forces of Poly‐CNCs_4wt.%_‐DN film with respect to varying pH, respectively.

The impact of pH on the chiroptical effects of the Poly‐CNCs_4wt.%_‐DN film were also investigated, as presented in **Figure** **5**. The CD and UV‐vis absorption spectra of the Poly‐CNCs_4wt.%_‐DN film exhibited significant changes with varying pH levels (Figure 5a). At pH 7, the CD signal was the strongest, indicating a stable left‐handed helical structure. As acidity increased, the CD signal intensity gradually decreased. At pH 6, 5, 4 and 3, the CD signal weakened significantly due to protonation disrupting the π─H bonds network and chiral structure. At pH 2 and 1, the CD signal further decreased, indicating a transition toward a racemic helical structure because of severe disruption of intermolecular π─H bonds interactions.^[^ ^32^ ^]^ Meanwhile, as acidity increased, CPL (Figure 5b; Table S5, Supporting Information) and PL (Figure 5e) signal intensities also decreased. At pH 6, CPL and PL signals slightly decreased as protonation begins influencing the electronic structure of the naphthalene rings. When the pH decreased from 6 to 3, the CPL and PL signals further decreased due to more pronounced protonation effects, which disrupted the conjugation system and hydrogen bond network of the naphthalene rings, leading to reduced fluorescence intensity. Under high acidity at pH 2 and 1, intermolecular π─H bonds were compromised, resulting in damage to the chiral structure and luminescent properties of the Poly‐CNCs_X_‐DN film.

As alkalinity increased (Figure 5c), the CD signal of Poly‐CNCs_4wt.%_‐DN film significantly declined at pH 8, with a slight decrease in the UV‐vis absorption peak. At pH 9, the CD signal reversed, accompanied by a slight blue‐shift in the UV‐vis absorption, indicating a transformation from a left‐handed to a right‐handed helical structure (Figure 5g),^[^ ^33^ ^]^ due to the deprotonation of ‐OH on CNCs surfaces. The deprotonation of ‐OH of CNCs resulted in the formation of negatively charged alkoxides, which altered the original π─H bonds network and caused a change in chiral structure. With an increase in pH from 9 to 14, the CD signal initially increased and then decreased, accompanied by a notable blue‐shift in the UV‐vis absorption peak, indicating that changes in electrostatic interactions and π─H bonds adjusted the mode of intermolecular interaction, leading to the formation of more inversion points and random fragments (Figure 5g). Moreover, at pH 8, the CPL (Figure 5d; Table S6, Supporting Information) and PL signals weakened significantly. In the pH range of 9 to 14, the CPL signal of the Poly‐CNCs_4wt.%_‐DN film reversed, initially increasing and then rapidly decreasing, due to the rearrangement of the π─H bonds network accompanied by more inversion points and random fragments. This was consistent with the CD spectra. In addition, the PL spectrum (Figure 5d) and fluorescent photograph (Figure 5e) showed decreasing luminescence intensity with increasing alkalinity. Under alkaline conditions, the π‐π stacking and conjugated system of the naphthalene rings were impaired, leading to a decrease in luminescence intensity and a blue‐shift in the emission wavelength. These results demonstrated that the chiral and luminescent properties of the Poly‐CNCs_4wt.%_‐DN film were highly sensitive to acidic environments, emphasizing the importance of controlling pH levels in applications involving chiral materials and responsive optical devices.

Hence, these Poly‐CNCs_X_‐DN films exhibited multimodal‐responsive CD, PL, and CPL effects under conditions such as temperature, solvent polarity, and pH. This multimodal responsiveness arose from the high sensitivity of the π─H bonds network in these chiral supramolecular RPU to external stimuli. These interactions led to a shortening of the helix persistence length and an increase in helical inversion sites, causing conformational transitions of the RPU chain from a left‐handed to a right‐handed helix or a random coil structure.

### 2.4 Flexible Display Application

**Figure 6:**
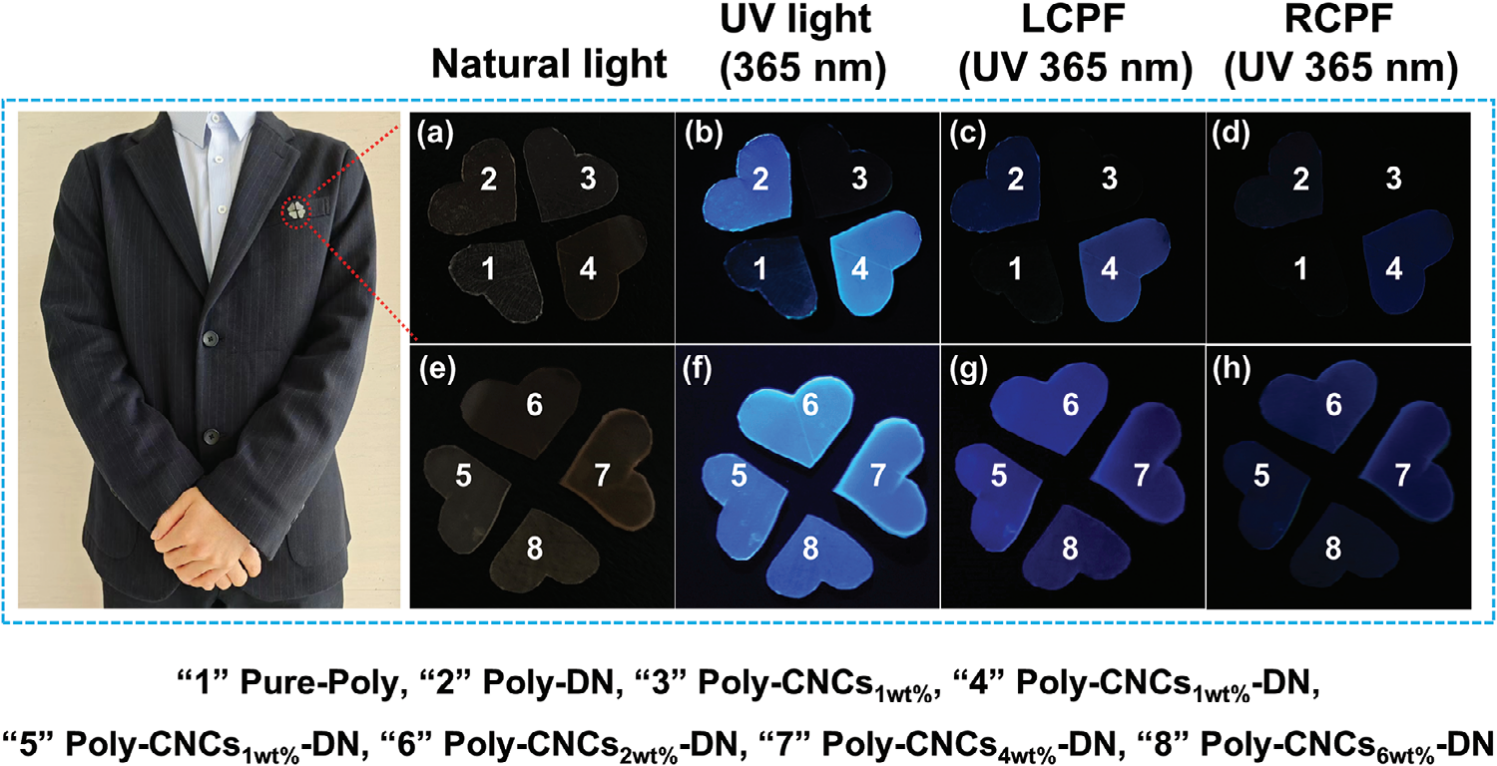
Evaluation on the anti‐counterfeiting capability. Optical photos of latent patterns constructed by Poly‐CNCs_X_‐DN films taken under different lighting conditions: a and e) natural light; b and f) UV light of 365 nm; c and g) UV light of 365 nm under a left‐handed circular polarizer filter; d and h) UV light of 365 nm under a right‐handed circular polarizer filter.

Poly‐CNCs_X_‐DN films exhibited remarkable circularly polarized luminescence (CPL) properties, making them highly suitable for information encryption applications. **Figure** **6** demonstrated polarization‐based encryption using Pure‐Poly, Poly‐DN, Poly‐CNCs_1wt.%_‐DN, Poly‐CNCs_2wt.%_‐DN, Poly‐CNCs_4wt.%_‐DN, Poly‐CNCs_6wt.%_‐DN films. These RPU films, directly formed on fabric, leverage their strong chemical adhesion to substrates with active hydrogen due to their high polarity and reactivity. The first four‐leaf clover shaped pattern in the upper layer of the Figure 6, crafted from Pure‐Poly (1), Poly‐DN (2), Poly‐CNCs_1wt.%_ (3) and Poly‐CNCs_1wt.%_‐DN (4) films, were displayed in Figure 6a–d. Under natural light, the white transparent color of first “four‐leaf clover” was visible against a black fabric (Figure 6a). Under 365 nm UV light, the “2” and “4” of first “four‐leaf clover” emitted blue fluorescence (Figure 6b), indicating a first‐level anticounterfeiting. When viewed through left and right circular polarizer filters, respectively, the fluorescence dimmed, with “2” and “4” appearing more prominent through the left circular polarizer filter (Figure 6d) than the right circular polarizer filter (Figure 6c), due to the left‐handed CPL activity of the Poly‐CNCs_1wt.%_‐DN film, establishing a secondary anti‐counterfeiting behavior.^[^ ^34^ ^]^ Notably, despite a low *g*_lum_ value (≈0.01), the Poly‐CNCs_X_‐DN films showed noticeable fluorescence intensity changes under right‐ and left‐handed circular polarizers. This can primarily be attributed to the long‐range chiral structure formed within the film, which enhanced the interaction between light and the material.^[^ ^35^ ^]^

To further assess the anti‐counterfeiting capabilities of Poly‐CNCs_X_‐DN films, the second four‐leaf clover shaped pattern in the lower layer of the Figure 6e–h, composed of Poly‐CNCs_1wt.%_‐DN (5), Poly‐CNCs_2wt.%_‐DN (6), Poly‐CNCs_4wt.%_‐DN (7), and Poly‐CNCs_6wt.%_‐DN (8), was evaluated under various lighting conditions. Under natural light, the white transparent color of second “four‐leaf clover” was visible against a black fabric (Figure 6e). Under 365 nm UV light, all Poly‐CNCs_X_‐DN films exhibited blue fluorescence of varying intensities (Figure 6f), demonstrating first‐level anti‐counterfeiting performance. The blue fluorescence from “5–8” diminished progressively under left (Figure 6g) and right circular polarizer filters (Figure 6h), with Poly‐CNCs_4wt.%_‐DN showing the strongest left‐handed CPL activity, indicative of secondary, wearable, flexible anti‐counterfeiting properties. These findings underscored the critical role of the main‐chain helical chirality in Poly‐CNCs_X_‐DN films in generating left‐handed CPL, positioning these CPL elastomer films as viable wearable, flexible security materials.

In addition, Figure S10 (Supporting Information) illustrated the mechanical performance and deformation behavior of Pure‐Poly, Poly‐DN, Poly‐CNCs_X,_ and Poly‐CNCs_X_‐DN films under tensile testing. The stress‐strain curves (Figure S10a, Supporting Information) showed that Pure‐Poly had a stress‐bearing capacity of ≈56 MPa and a fracture strain of ≈87%. Following the addition of 1,5‐DN, the strength and fracture strain of the Poly‐DN film increased to ≈67 MPa and 88%, respectively, attributed to the rigidity imparted by the naphthalene ring structure of 1,5‐DN. The introduction of 1 wt.% CNCs enhanced the mechanical strength of the Poly‐CNCs_1wt.%_ film compared to the Pure‐Poly film, likely due to hydrogen bond cross‐linking. The combination of CNCs and 1,5‐DN further improved mechanical strength and toughness of the Poly‐CNCs_X_‐DN film, suggesting the formation of a more ordered cross‐linked network by π─H bonds between CNCs and naphthalene rings. The Poly‐CNCs_4wt.%_‐DN film achieved an optimal balance of stress (91 MPa) and strain (105%), maximizing reinforcement and cross‐linking effects. Conversely, the Poly‐CNCs_6wt.%_‐DN film showed decreased toughness due to excessive cross‐linking and hard segment aggregation. The deformation images (Figure S10b, Supporting Information) demonstrated the flexibility and recovery of Poly‐CNCs_X_‐DN films, as it can nearly return to its original shape after deformation, indicating excellent elastic recovery. This property made it promising for applications requiring repeated mechanical stress, such as flexible displays and advanced functional materials with robust mechanical properties.

Subsequently, we carried out a tensile responsivity test of the Poly‐CNCs_4wt.%_‐DN film (Figure S11, Supporting Information). As the elongation of Poly‐CNCs_4wt.%_‐DN increased, a gradual decrease in the CPL signal was observed, attributable to the decreased concentration of CNCs and 1,5‐DN per unit area in the stretched Poly‐CNCs_4wt.%_‐DN film.^[^ ^36^ ^]^ These above results collectively demonstrated the changes in mechanical and optoelectronic properties of the material under different strain conditions. These results provided important design insights for developing high‐performance RPU materials that combine flexibility and optoelectronic functions.

Overall, RPU enabled high‐adhesion in situ polymerization on flexible substrates, forming a highly crosslinked and stable film without solvent evaporation, which integrated flexibility, environmental responsiveness and optical signal modulation. Its crosslinked network governed by hydrogen bonding, allowed reversible reconfiguration under light, heat and mechanical stimuli, enabling information concealment and precise anti‐counterfeiting control. CNCs, through helical self‐assembly, exhibited chiral amplification, enhanced CPL and dynamic responsiveness, which ensured optical stability under humidity, temperature and mechanical stress, thus enhancing the recognition capability and environmental adaptability of flexible anti‐counterfeiting materials.

## 3 Conclusion

In summary, we report a novel solvent‐free supramolecular polymerization self‐assembly strategy for chiral supramolecular polymer materials, based on the additive polymerization of liquid CNCs‐based isocyanate prepolymers and liquid polyols (PTMEG that is dissolved with 1,5‐DN), achieving ordered and controllable assembly of polymer chains. This approach constructs CPL flexible RPU (Poly‐CNCs_X_‐DN) films with feather‐like nanostructures by incorporating CNCs and 1,5‐DN as chiral and fluorescent units, repectively, promoting the formation of the π─H bonds network between the CNCs and the multiple benzene rings linked by urethane groups, and driving the ordered arrangement of CNCs. Furthermore, these Poly‐CNCs_X_‐DN films exhibit multimodal‐responsive CD, PL, and CPL effects under conditions such as temperature, solvent polarity, pH, and polarized light stimulation. This is because the π─H bonds network is susceptible to external stimuli, causing the Poly‐CNCs_X_‐DN main chain to undergo a conformational transition from a left helix to a right helix or random coil structure. Therefore, our work can promote not only the development of smart materials based on the stimuli‐responsive cascade processes but also the design of novel multi‐channel chiroptical flexible materials.

## Conflict of Interest

The authors declare no conflict of interest.

## Supporting information



Supporting Information

## Data Availability

The data that support the findings of this study are available in the supplementary material of this article.
