# Supplementary material for: Solvent‐Free Supramolecular Polymerization for Feather‐Like Nanostructured Chiral Fluorescent Polyurethanes with Multimodal Chiroptical Stimuli Responsiveness
Source: Adv Sci (Weinh). 2025 Feb 28;12(16):2417572. doi: 10.1002/advs.202417572 (PMC12021037; doi:10.1002/advs.202417572)
Supplement: Supplementary file 1 — Supporting Information [file ADVS-12-2417572-s001.docx]

Supporting Information

**Solvent-free supramolecular polymerization for feather-like nanostructured chiral fluorescent polyurethanes with multimodal chiroptical stimuli responsiveness**

*Huimin Duan ^a,c,d,#^*,* *Shuli Li ^a,#^,* *Xinlei Wu ^a,c^,* *Jianping Deng ^b^,* *Jiawei Li ^a^*, Dongming Qi ^a,c^*,* *Biao Zhao^b^**

Dr. H. M. Duan, S. L. Li, Dr. X. L. Wu, Dr. J. W. Li, Prof. D. M. Qi
School of Textile Science and Engineering, School of Materials Science and Engineering & School of Chemistry and Chemical Engineering, Zhejiang Sci-Tech University, Zhejiang 310018, China.
E-mail: 201820302008@mails.zstu.edu.cn, jiaweili@zstu.edu.cn, dongmingqi@zstu.edu.cn.

Prof. J. P. Deng, Prof. B. Zhao
State Key Laboratory of Chemical Resource Engineering, College of Materials Science and Engineering, Beijing University of Chemical Technology, Beijing 100029, China.
E-mail: zhaobiao@mail.buct.edu.cn

Dr. H. M. Duan, Dr. X. L. Wu, Prof. D. M. Qi
Zhejiang Provincial Innovation Center of Advanced Textile Technology, Zhejiang 312000, China.

Dr. H. M. Duan
Keqiao Research Institute of Zhejiang Sci-Tech University, Zhejiang 312000, China.

**Contents**

**Figure S1.** Synthetic route of chiral fluorescent CNCs-based -NCO prepolymers.

**Figure S2.** Synthetic route of chiral fluorescent Poly-CNCs_X_-DN films.

**Figure S3.** Optical images of CNCs (a. the suspension after dialysis; b. light passes through the suspension after dialysis; c. freeze-dried powder).

**Figure S4.** SEM images (a) of CNCs and MCCs; POM images (b) and FT-IR (c) spectra of CNCs.

**Figure S5.** XRD patterns of CNCs and MCCs.

**Figure S6.** Solubility images of Poly-CNCs_4wt%_-DN film in common solvents.

**Figure S7.** Optical images of Pure-Poly, Poly-DN, Poly-CNCs_1wt%_ and Poly-CNCs_X_-DN films with DMF treatment for 6 h.

**Figure S8.** CPL spectra of Poly-CNCs_4wt%_-DN film with different geometric testing angles.

**Figure S9.** CD spectra of Poly-CNCs_1wt%_ film and fluorescence spectra of Poly-DN film.

**Figure S10.** Tensile stress-strain curves (a) of Poly-CNCs_X_-DN films. Stretching, torsion, folding and flexure behavior under external forces (b) of Poly-CNCs_4wt%_-DN film.

**Figure S11.** CPL spectra of Poly-CNCs_4wt%_-DN film with different tensile deformations.

**Table S1.** List of synthetic ingredients for Pure-Poly, Poly-DN, Poly-CNCs_1wt%_, and Poly-CNCs_X_-DN films.

**Table S2.** Photophysical parameters of Poly-CNCs_X_-DN films.

**Table S3.** Temperature-responsive *g*_lum_ value of CPL performance for Poly-CNCs_4wt%_-DN film.

**Table S4.** Solvent polarity-responsive *g*_lum_ value of CPL performance for Poly-CNCs_4wt%_-DN film.

**Table S5.** pH (acidity)-responsive *g*_lum_ value of CPL performance for Poly-CNCs_4wt%_-DN film.

**Table S6.** pH (alkalinity)-responsive *g*_lum_ value of CPL performance for Poly-CNCs_4wt%_-DN film.

**Experimental Section**

*Materials*: The microcrystalline cellulose (MCCs, column chromatography) with average particle size μm, 2,4-toluene diisocyanate (TDI, 98%), 1,5-dihydroxy naphthalene (1,5-DN, 98%), 1,4-butanediol (BDO, analytical grade), and polytetrahydrofuran ether diol (PTMEG-1000, *M*_n_=1000 g/mol) were purchased from Aladdin. Concentrated sulfuric acid (98% mass fraction, H_2_SO_4_) was purchased from Nanning Yuanlai instrument Co. Ltd. PTMEG was vacuum-dried at 100 °C for approximately 12 h before use, while the other reagents were used as received.

*Measurements*: Attenuated total reflectance (ATR)-Fourier transform infrared (FTIR) spectra were recorded using a Nicolet 5700 infrared spectrometer (Thermal Power Company, USA). The crystallinity was examined by X-ray diffraction (XRD) using a Bruker D8 diffractometer with a Cu Kα radiation source, with a wavelength of 0.15418 nm, accelerating voltage 40 kV, accelerating current 40 mA, scanning speed 5° min^−1^, and a 2θ angle range of 5°-50°. Raman spectra were obtained on a LabRAM HR Evolution instrument (Horiba Scientific, Japan) under laser pulses at *λ* = 325 nm. Scanning electron microscopy (SEM) analyses were performed on a GeminiSEM500 (Zeiss, UK). Polarized optical microscopy (POM) was performed using an Axioskop 40A Pol optical microscope (Carl Zeiss). Atomic force microscopy (AFM) images were obtained on a Bruker Dimension Icon with the tapping mode. Circular dichroism (CD), and ultraviolet (UV)–visible (vis) absorption spectra were measured using a DSM 172 spectrophotometer (OLIS, USA) equipped with a Peltier light source and a water-circulating temperature controller. Circularly polarized luminescence (CPL, excited at 365 nm) were performed using a CPL-300 (JASCO, Japan). Fluorescence spectra were collected on a F-4600 spectrophotometer (Hitachi, Japan). Thermogravimetric (TG) was conducted using a TG209 F1 Libra thermogravimetric analyzer (NETZSCH, Germany) under a nitrogen atmosphere, 5-6 mg of a sample was tested at a heating rate of 10 °C min^-1^ within the range of 30-800 °C.

*Preparation of CNCs*: Concentrated sulfuric acid (98%) was diluted to 64% with deionized water. In a three-necked flask, 10 grams of microcrystalline cellulose (MCCs) were added to 100 milliliters of 64% sulfuric acid, and the mixture was vigorously stirred at 700 rpm for 45 min at 45 °C to complete the hydrolysis. Following the reaction, ten times the volume of deionized water was added to terminate the reaction. The solution was allowed to stand for 12 h, after which the clarified liquid was decanted. The resulting suspension was centrifuged three to five times until the supernatant appeared as a blue and clear colloid. Subsequently, the solution was placed in a dialysis membrane tube with a molecular weight cutoff of 10,000 and dialyzed for one week until the pH of the solution reached neutrality or the electrical conductivity approached that of deionized water, yielding a light blue colloidal solution (**Figure S3a**). After dialysis, the solution was freeze-dried to obtain a white powder (**Figure S3c**).

*Preparation of chiral fluorescent CNCs-based -NCO prepolymers*: TDI and PTMEG-1000 dissolved with 1,5-DN were mixed with a specified amount of CNCs (accounting for 1%, 2%, 4%, and 6% of the total mass of the system) and subjected to a polyaddition reaction in an oil bath at 80 °C with mechanical stirring at 400 rpm under a nitrogen atmosphere. The experiment was halted once the -NCO content in the system, measured by dibutylamine titration, reached the theoretical value of 30%, resulting in the formation of chiral fluorescent CNCs-based -NCO prepolymer (Component B). The prepolymers of Pure-Poly, Poly-CNCs_X_, and Poly-DN for the control groups were synthesized using the same procedures. The detailed synthetic route is illustrated in **Figure S1**.

*Preparation of chiral fluorescent supramolecular polyurethane (Poly-CNCs_X_-DN)*: A certain proportion of 1,4-BDO was mixed homogeneously and used as a small molecule chain extender. After that, polyol PTMEG-1000 was added into the above small molecule chain extender and stirred homogeneously at room temperature, resulting in a polyhydric alcohol mixture (component A). The mixed component A and component B were stirred for 5 min under vacuum, and the obtained liquid was placed in PTFE molds and aged at high temperature of 120 ^o^C for 24 h reaction as shown in **Figure S2**. The chiral fluorescent supramolecular polyurethane were prepared by solvent-free component in-situ polymerization with 1 wt%, 2 wt%, 4 wt% and 6 wt% of CNCs, which were named as Poly-CNCs_1wt%_-DN, Poly-CNCs_2wt%_-DN, Poly-CNCs_4wt%_-DN, and Poly-CNCs_6wt%_-DN. **Table S1** and **Figure S2** presented the detailed synthetic contents and routes, respectively, for the Poly-CNCs_X_-DN films, as well as for the control groups: Pure-Poly, Poly-CNCs_X_, and Poly-DN films.

**Figure S1.** Synthetic route of chiral fluorescent CNCs-based -NCO prepolymers.

**Figure S2.** Synthetic route of chiral fluorescent Poly-CNCs_X_-DN films.


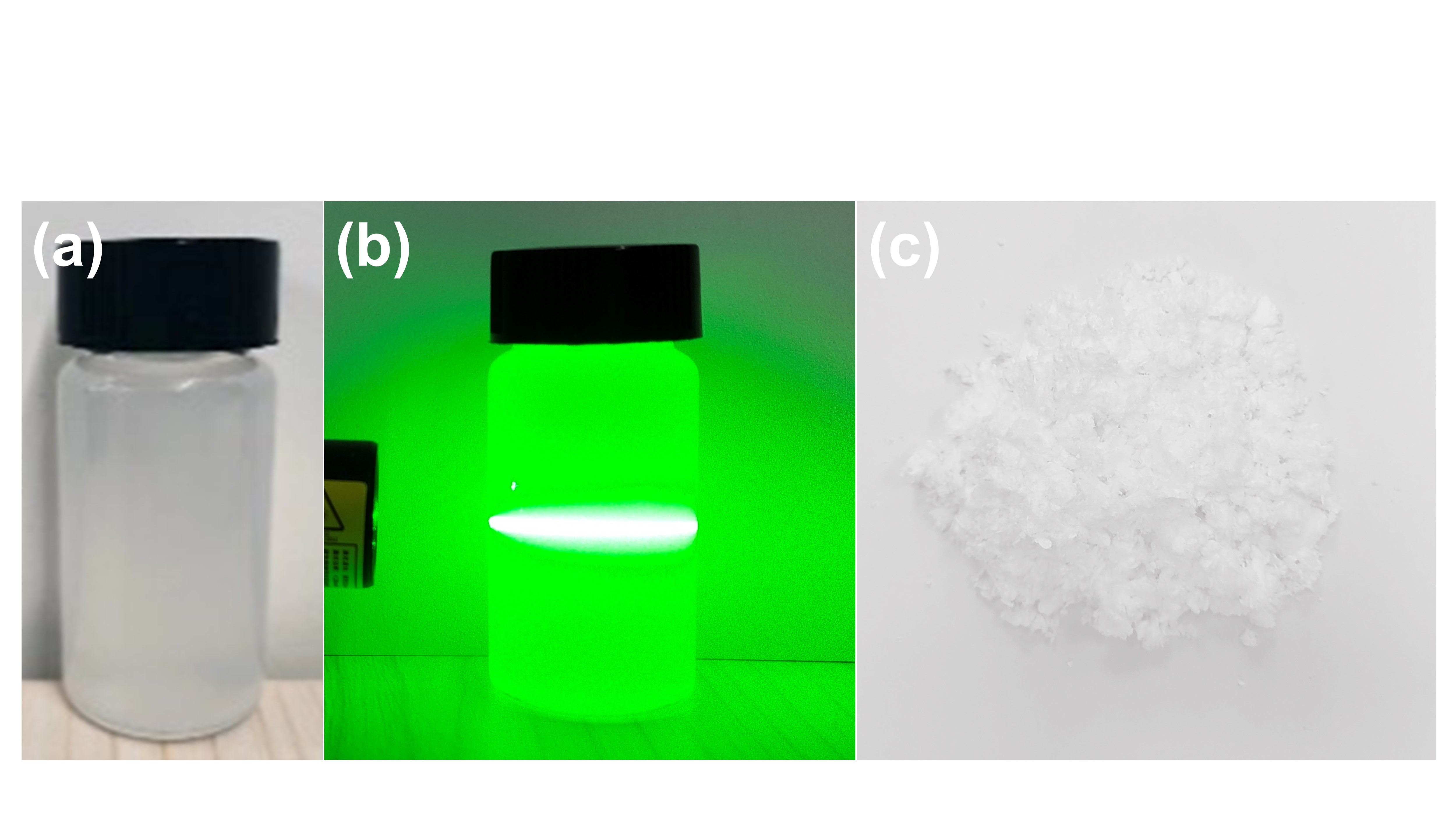


**Figure S3.** Optical images of CNCs (a. the suspension after dialysis; b. light passes through the suspension after dialysis; c. freeze-dried powder).


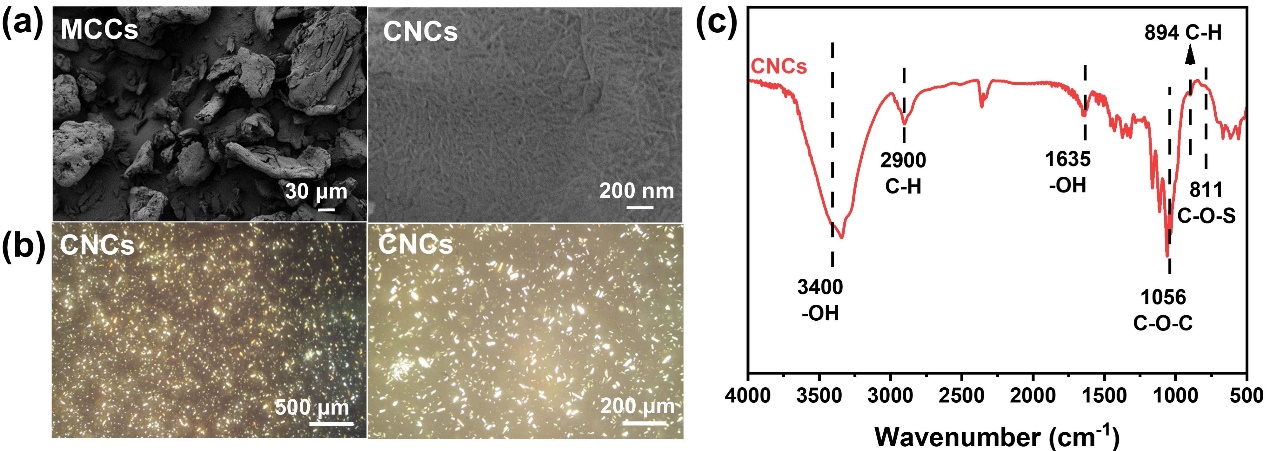


**Figure S4.** SEM images (a) of CNCs and MCCs; POM images (b) and FT-IR (c) spectra of CNCs.

**Figure S5.** XRD patterns of CNCs and MCCs.

**
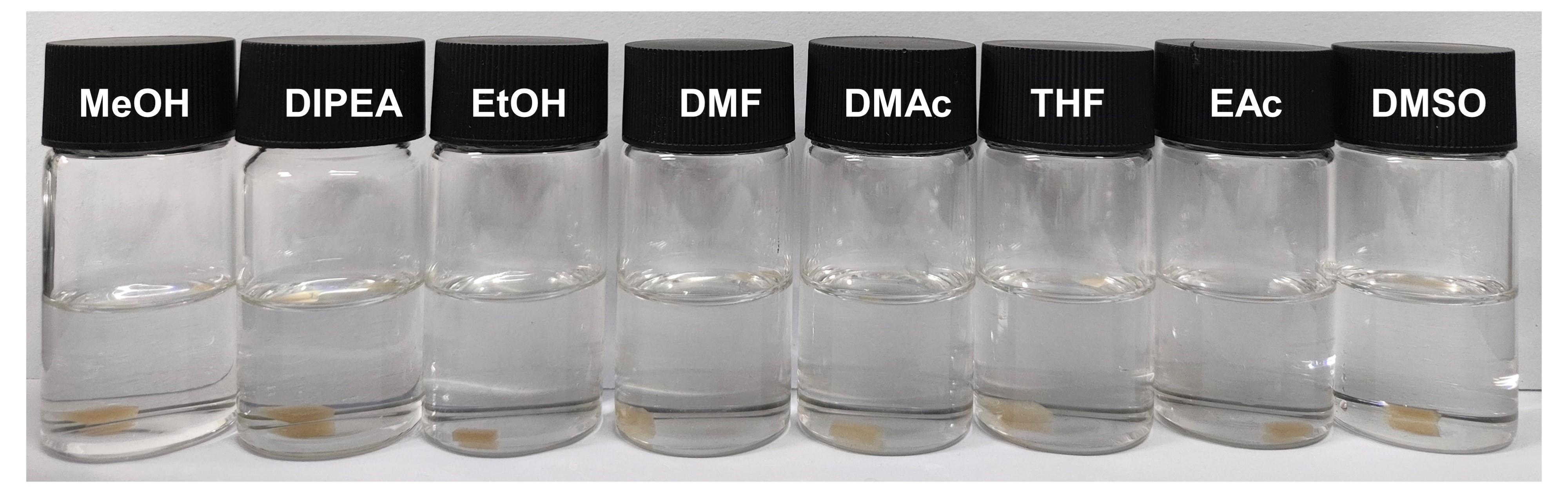
**

**Figure S6.** Solubility images of Poly-CNCs_4wt%_-DN film in common solvents.


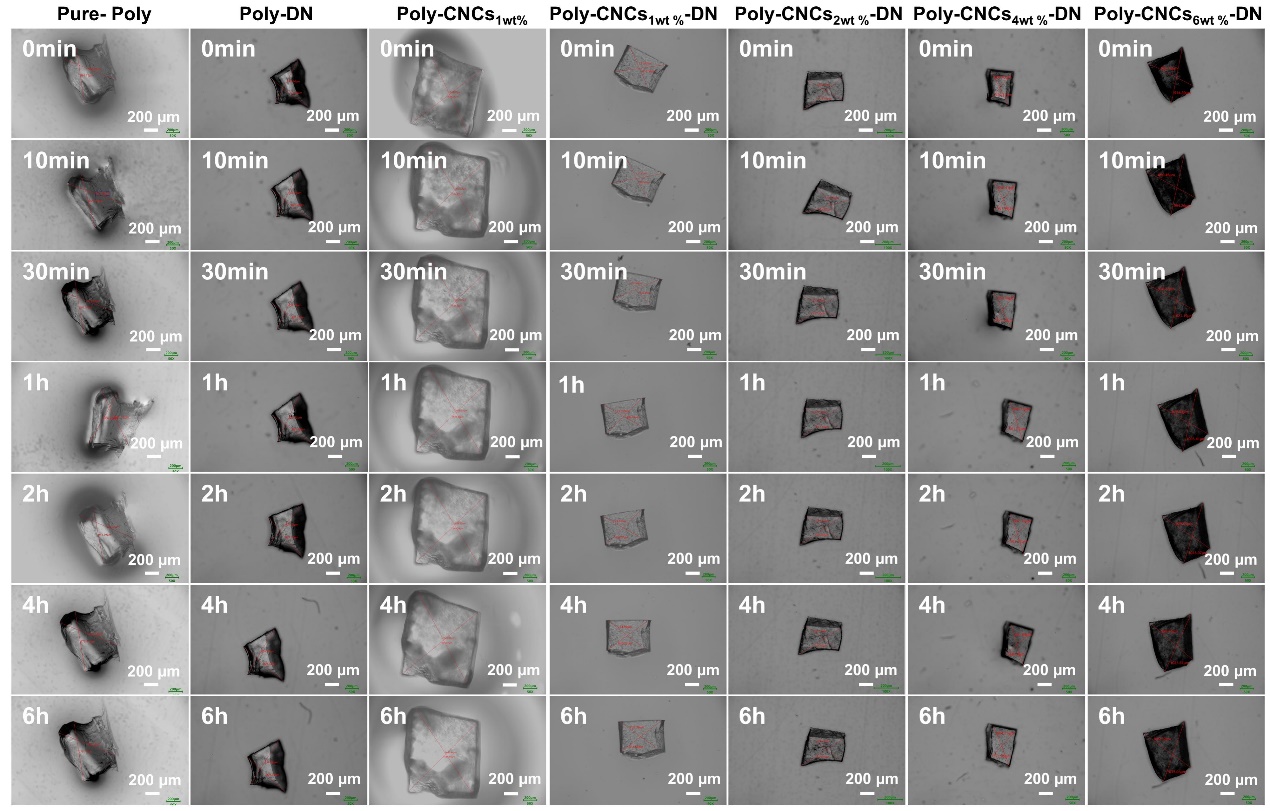


**Figure S7.** Optical images of Pure-Poly, Poly-DN, Poly-CNCs_1wt%_ and Poly-CNCs_X_-DN films with DMF treatment for 6 h.

**Figure S8.** CPL spectra of Poly-CNCs_4wt%_-DN film with different geometric testing angles.

**
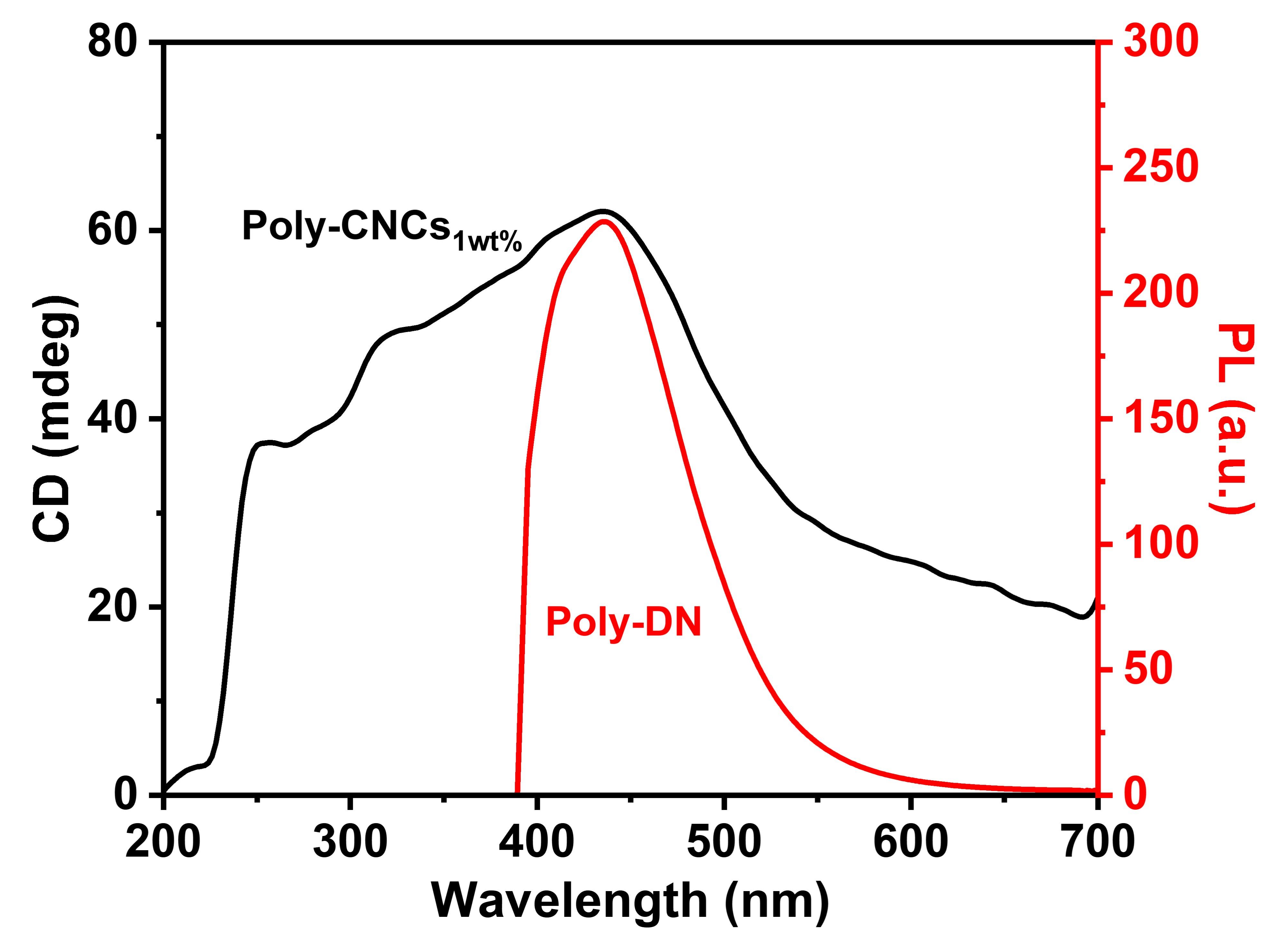
**

**Figure S9.** CD spectra of Poly-CNCs_1wt%_ film and fluorescence spectra of Poly-DN film.


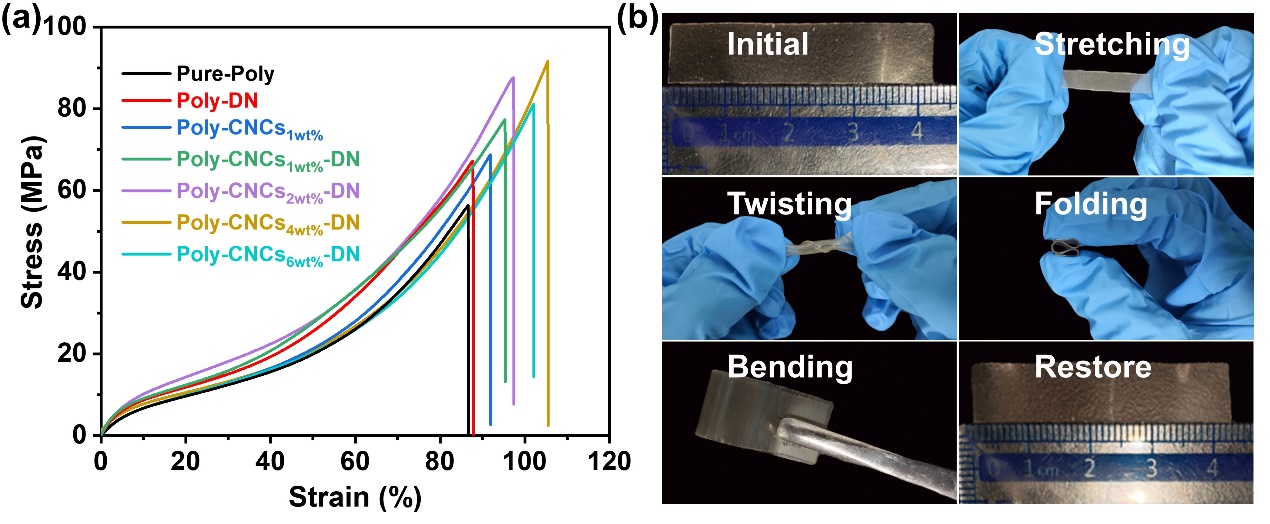


**Figure S10.** Tensile stress-strain curves (a) of Poly-CNCs_X_-DN films. Stretching, torsion, folding and flexure behavior under external forces (b) of Poly-CNCs_4wt%_-DN film.

**Figure S11.** CPL spectra of Poly-CNCs_4wt%_-DN film with different tensile deformations.

**Table S1.** List of synthetic ingredients for Pure-Poly, Poly-DN, Poly-CNCs_1wt%_, and Poly-CNCs_X_-DN films.

| Sample | Component B (g) | | | |  | | Component A (g) | |
| --- | --- | --- | --- | --- | --- | --- | --- | --- |
|  | TDI | CNCs | PTMEG | 1,5-DN |  | BDO | | PTMEG |
| Pure-Poly | 21.06 | 0 | 10 | 0 |  | 3.17 | | 46.89 |
| Poly-DN | 21.06 | 0 | 10 | 0.12 |  | 3.17 | | 46.89 |
| Poly-CNCs_1wt%_ | 21.06 | 0.31 | 10 | 0 |  | 3.17 | | 46.89 |
| Poly-CNCs_1wt%_-DN | 21.06 | 0.31 | 10 | 0.12 |  | 3.17 | | 46.89 |
| Poly-CNCs_2wt%_-DN | 21.06 | 0.62 | 10 | 0.12 |  | 3.17 | | 46.89 |
| Poly-CNCs_4wt%_-DN | 21.06 | 1.24 | 10 | 0.12 |  | 3.17 | | 46.89 |
| Poly-CNCs_6wt%_-DN | 21.06 | 1.86 | 10 | 0.12 |  | 3.17 | | 46.89 |

**Table S2.** Photophysical parameters of Poly-CNCs_X_-DN films.

| Sample | Emission wavelength (nm) | Quantum yields (*Φ*_F_/%) | Fluorescence lifetimes (*τ*/ns) | *ε_λ_*  (M^-1^  cm^-1^) | \|*g*_lum_\| | CPL brightness (*B*_CPL_/M^-1^cm^-1^) |
| --- | --- | --- | --- | --- | --- | --- |
| Poly-DN | 448 | 1.44 | 5.66 | / | / | / |
| Poly-CNCs_1wt%_ | / | / | / | 38_330nm_ | / | / |
| Poly-CNCs_1wt%_-DN | 450 | 5.08 | 6.39 | 129_334nm_ | 0.0035 | 1.15 |
| Poly-CNCs_2wt%_-DN | 452 | 7.66 | 8.18 | 214_349nm_ | 0.0066 | 5.41 |
| Poly-CNCs_4wt%_-DN | 453 | 8.80 | 9.92 | 360_355nm_ | 0.0115 | 18.22 |
| Poly-CNCs_6wt%_-DN | 452 | 8.17 | 9.10 | 296_360nm_ | 0.0075 | 9.07 |

**Table S3.** Temperature-responsive *g*_lum_ value of CPL performance for Poly-CNCs_4wt%_-DN film.

| Temperature (℃) | 20 | 30 | 40 | 50 | 60 | 70 | 80 | 90 | 90-20 |
| --- | --- | --- | --- | --- | --- | --- | --- | --- | --- |
| *\|g*_lum_\| | 0.0115 | 0.0102 | 0.0084 | 0.0067 | 0.0058 | 0.0048 | 0.0039 | 0.0023 | 0.0092 |

**Table S4.** Solvent polarity-responsive *g*_lum_ value of CPL performance for Poly-CNCs_4wt%_-DN film.

| Solvent polarity | None | DCM | EtOH | MeOH | DMF | DMSO |
| --- | --- | --- | --- | --- | --- | --- |
| *\|g*_lum_\| | 0.0115 | 0.0079 | 0.0051 | 0.0040 | 0.0038 | 0.0032 |

**Table S5.** pH (acidity)-responsive *g*_lum_ value of CPL performance for Poly-CNCs_4wt%_-DN film.

| pH | 1 | 2 | 3 | 4 | 5 | 6 | 7 |
| --- | --- | --- | --- | --- | --- | --- | --- |
| *\|g*_lum_\| | 0.0002 | 0.0004 | 0.0056 | 0.0077 | 0.0087 | 0.0092 | 0.0115 |

**Table S6.** pH (alkalinity)-responsive *g*_lum_ value of CPL performance for Poly-CNCs_4wt%_-DN film.

| pH | 7 | 8 | 9 | 10 | 11 | 12 | 13 | 14 |
| --- | --- | --- | --- | --- | --- | --- | --- | --- |
| *g*_lum_ | +0.0115 | +0.0031 | -0.0044 | -0.0048 | -0.0058 | -0.0059 | -0.0031 | -0.0025 |
